# Stabilization of a bat-pitcher plant mutualism

**DOI:** 10.1038/s41598-017-13535-5

**Published:** 2017-10-13

**Authors:** Michael G. Schöner, Caroline R. Schöner, Rebecca Ermisch, Sébastien J. Puechmaille, T. Ulmar Grafe, Moi Chan Tan, Gerald Kerth

**Affiliations:** 1 Zoological Institute and Museum, University of Greifswald, Loitzer Straße 26, 17489 Greifswald, Germany; 2 Faculty of Science, Biology, University Brunei Darussalam, Tungku Link, Gadong, 1410 Brunei Darussalam

## Abstract

Despite the long persistence of many mutualisms, it is largely unknown which mechanisms stabilize these interactions. This is especially true if only one mutualism partner can choose alternative partners while the other cannot, resulting in a power asymmetry. According to biological market theory the choosing partner should prefer the more dependent partner if the latter offers commodities of higher quality than its competitors. We tested this prediction using Bornean carnivorous pitcher plants (*Nepenthes hemsleyana*) that strongly rely on faecal nitrogen of bats (*Kerivoula hardwickii*) which roost inside the pitchers. The bats also roost in furled leaves of various plants. Surprisingly, during field observations the bats did not always choose *N. hemsleyana* pitchers despite their superior quality but were generally faithful either to pitchers or to furled leaves. In behavioural experiments 21% of the leaf-roosting bats switched to pitchers, while the majority of these bats and all pitcher-roosting individuals were faithful to the roost type in which we had found them. Genetic differentiation cannot explain this faithfulness, which likely results from different roosting traditions. Such traditions could have stabilizing or destabilizing effects on various mutualisms and should be investigated in more detail.

## Introduction

Despite their importance and ubiquity, “the evolution and maintenance of mutualisms remains a largely unsolved puzzle”^1^. Clear evidence exists that mutualisms evolved and disappeared repeatedly and notably shifted from autonomy to mutualism and vice versa^2–4^. Recent findings however indicate that mutualisms are not as unstable as previously thought^5^. Empirical research on the stabilization of mutualisms is generally rare, has mostly focused on obligate mutualisms (e.g., between figs and fig wasps^1^) and contradicts theoretical models^6^. In facultative mutualisms where partners have asymmetric power in choosing alternative partners, it is generally assumed that the quality of the partners is crucial for the stabilization of the interaction. Biological market theory (BMT) predicts that individuals which can choose between alternative interaction partners should select partners of highest quality. Accordingly, individuals of the more dependent species should outperform their intra- and interspecific competitors by offering commodities of higher quality^7–9^. However, studies show that not only the choice of high quality partners stabilizes interactions but also alternative behavioural patterns such as partner control mechanisms, where one partner reacts to defections of the other e.g., via termination of the cooperation^10^.

Here, we empirically investigated the stabilization of an animal-plant mutualism: Woolly bats (*Kerivoula hardwickii*) fertilize carnivorous pitcher plants of the species *Nepenthes hemsleyana* with their faeces while using the plants’ pitcher-shaped trapping organs as roosts^11–13^. The plants strongly rely on the bats because arthropod capture is insufficient for *N. hemsleyana*’s nutrient demand^14^. In contrast, the bats depend less on *N. hemsleyana* pitchers and also roost in dead pitchers of *Nepenthes bicalcarata* and *Nepenthes ampullaria* as well as furled leaves of plants in the families Araceae, Musaceae and Zingiberaceae^15,16^. These plants most likely do not profit to such an extent from the bats’ presence.

Only *N. hemsleyana* pitchers have acquired traits that are highly attractive for the bats. This includes a shape that perfectly fits to the bats’ body size^11,13^ and a more stable pitcher microclimate than in other *Nepenthes* species^12^. Most important, due to an effective echo-reflecting structure, *N. hemsleyana* pitchers can easily be detected and identified by the bats in the dense vegetation of their habitat^17,18^. Moreover, several adaptations of *N. hemsleyana* plants to capturing arthropods are also beneficial for the bats. The inner wall of *N. hemsleyana* pitchers is covered by a waxy layer^19,20^, which does not allow arthropods to place their eggs, larvae or pupae there^12^. Consequently, those bat individuals that exclusively roost in *N. hemsleyana* pitchers are free of certain ectoparasites that depend on the bats’ roost for parts of their development, need to switch roosts less often and are in a better body condition than individuals using alternative pitcher plant roosts^12^. Finally, *N. hemsleyana* pitchers are available on more successive days than furled leaves^21,22^.

Due to their use of various roost plants, *K. hardwickii* individuals should be unreliable mutualism partners for *N. hemsleyana* plants so that it is unclear how this mutualism is stabilized. Potentially, two mechanisms stabilize markets with two or more interacting partners: First, in economy, markets should be stable if supply and demand determine the prize for which a given quantity of a commodity is traded (as predicted by Walrasian law). However, in a biological context such market clearing is of low relevance^9^. The second and much more important mechanism for the stabilization of biological markets can be seen in processes that enable individuals of both mutualism partners to gain maximal benefits from their interaction, such as (co-) evolution^7–9^.

We hypothesized that a behavioural trait of the bats with potential evolutionary consequences stabilizes the mutualistic interaction with plants of the species *N. hemsleyana*: the bats’ choosiness in respect to roosts. We predicted that bat individuals will always prefer roosts with the highest quality (c.f.^7–9^), i.e. *N. hemsleyana* pitchers, which would make them faithful mutualism partners. This could explain why *N. hemsleyana* plants have so strongly specialized on bats in terms of nutrient demands and in terms of roost quality supply^12,14^.

Because of the possibility for experimental manipulations, this bat-pitcher plant interaction is a candidate system to reveal how partner selection potentially stabilizes mutualisms over evolutionary time scales. Such stabilizing mechanisms may be similarly found in other animal-plant mutualisms, but so far are largely undocumented.

## Results

### Which roosts do bats select under natural conditions?

Using radio-telemetry and passive integrated transponder (PIT) tags to individually mark bats we monitored *K. hardwickii* individuals roosting in *Nepenthes* pitchers (n = 174 bats) or furled leaves (n = 152 bats) in 10 different study sites in Brunei Darussalam and Sarawak/Malaysia for 30 ± 18 (mean ± s.d.) days per site (Supplementary Table S1). In two out of 10 study sites only furled leaves were present. Individuals living in the remaining eight study sites additionally could use pitchers of different *Nepenthes* species. In these sites, abundance of the available roost types/species generally did not seem to influence roost selection as the bats disproportionately used the occurring *N. hemsleyana* pitchers. In study site “Long Iman”, e.g., *N. hemsleyana* pitchers only made up 5% of all available roosts. The bats clearly preferred these pitchers and occupied all of them every day. In contrast, 38% of the available potential roosts were furled leaves, in which we have never found bats in that study site.

In altogether seven study sites where both roost types co-occurred, including “Long Iman”, individual bats only roosted in pitchers (six sites) or furled leaves (one site; Supplementary Table S1). Only at study site „Airport“ bats used both roost types. In this site we marked 43 bats with PIT-tags or followed them via radio-telemetry (over 3.70 ± 3.20 days on average, range: 1–14 days). During that time none of the monitored individuals switched between pitchers (seven individuals) and furled leaves (36 individuals).

### Which roosts do the bats select under controlled conditions?

**Figure 1:**
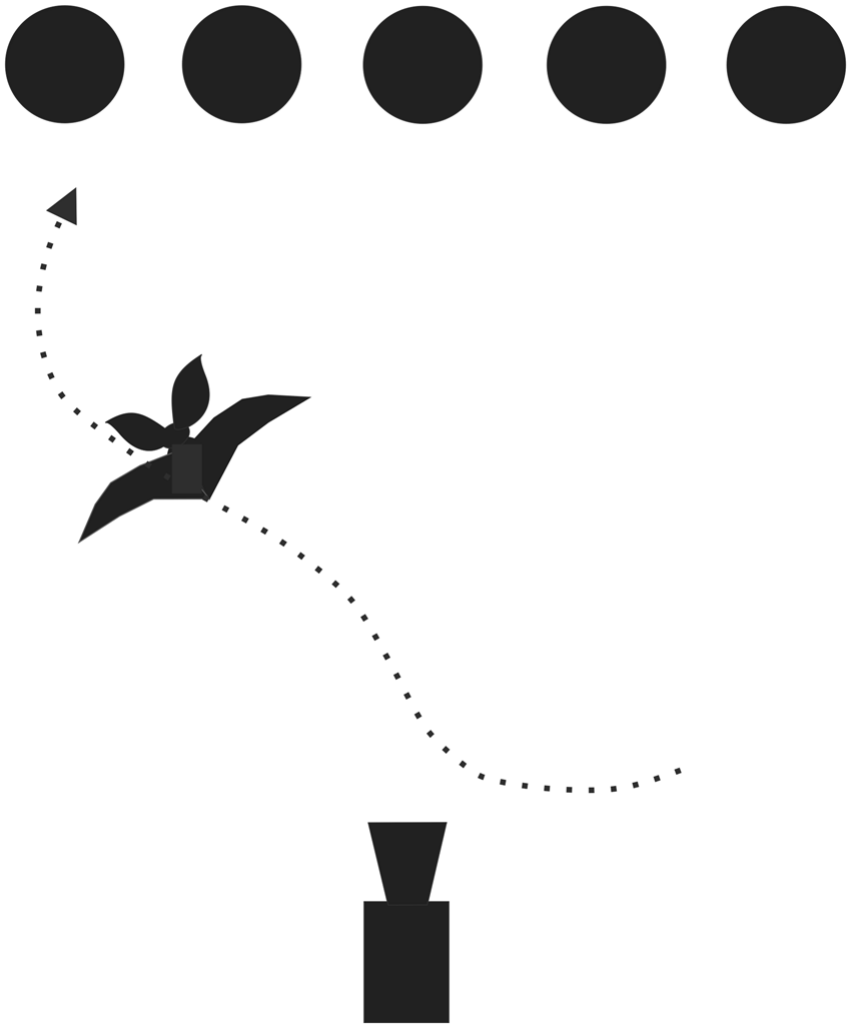
Experimental set-up of the behavioural experiments. Depending on the experiment we offered three to five potential roost types to the bats in a randomized linear array (full circles) (image drawn by C. Schöner).

In a series of behavioural experiments, we investigated whether the bats are faithful to one roost type (pitcher versus furled leaf) or even plant species or whether they have a general preference for *N. hemsleyana* pitchers. In a flight arena, we offered different potential plant roosts to the bats that we had randomly arranged for each trial (Fig. 1). As an indication for roost preference we took the bats’ number of approaches (Supplementary Results) to a roost and the final roost selection into account. We also scored whether an individual’s roost choice was independent of the roost type in which it had been found in the wild.

**Figure 2:**
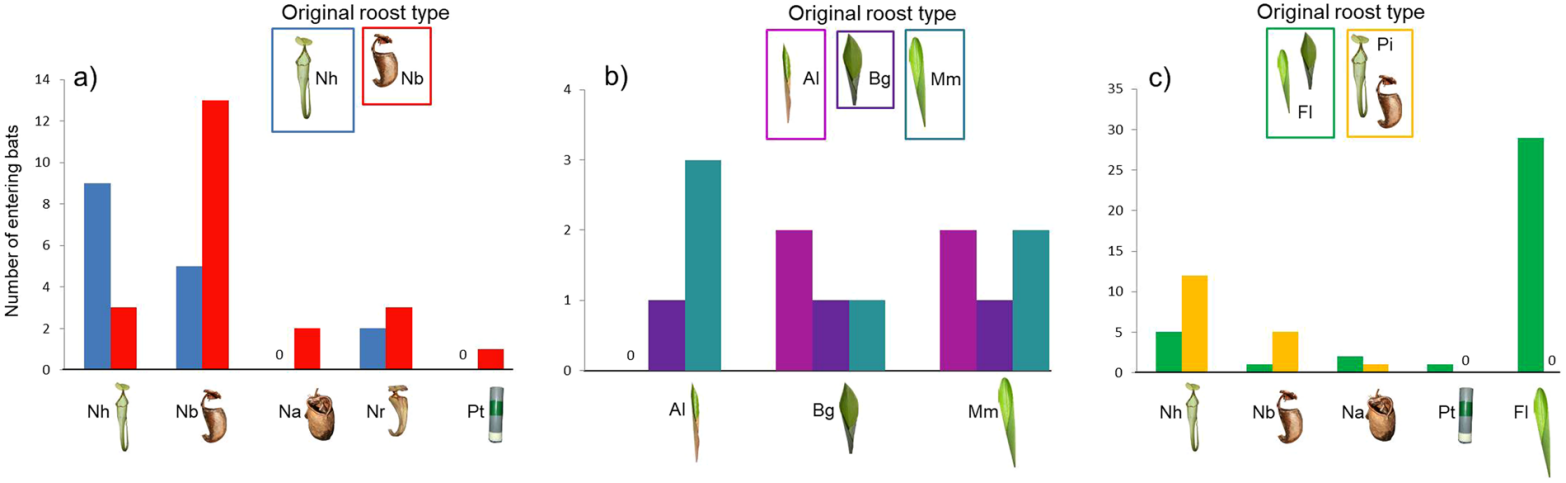
Roost preferences of *Kerivoula hardwickii* that used different roosts in the wild ( = original roost type). (**a**) Bats found in *Nepenthes hemsleyana* (Nh) or *Nepenthes bicalcarata* (Nb) could choose between pitchers of different *Nepenthes* species (*N. hemsleyana*, *N. bicalcarata*, *N. ampullaria* (Na), *N. rafflesiana* (Nr)) and a plastic tube (Pt). (**b**) Bats found in furled leaves of *Alpinia ligulata* (AI), *Boesenbergia grandis* (Bg) or *Musa muluensis* (Mm) could choose between furled leaves of these three species. (**c**) Bats found in furled leaves (Fl) or pitchers (Pi) could choose between different roost types (*N. hemsleyana*, *N. bicalcarata*, *N. ampullaria*, one furled leaf and the plastic tube; images created by C. and M. Schöner).

First, we tested whether the bats prefer pitchers of certain *Nepenthes* species to others. Forty-one bats from areas where *K. hardwickii* individuals only use *N. hemsleyana* (16 bats) and/or *N. bicalcarata* pitchers (25 bats) could choose between one *N. hemsleyana*, one *N. bicalcarata*, one *N. ampullaria* pitcher, one *Nepenthes rafflesiana* pitcher (which is not used by the bats), and a plastic tube with a similar size as the pitchers (width: 4.5 cm, length: 18.5 cm). The experiments showed that bats tended to be faithful to the pitcher plant species (*N. hemsleyana* or *N. bicalcarata*) in which we had originally found them in the wild (Table 1, Fig. 2a, Supplementary Results).

**Table 1:** Post hoc test results (Fisher’s exact tests for count data) of the behavioural experiments in the flight tent.

	Nh vs. Nb	Nh vs. Na	Nh vs. Nr	Nh vs. Pt	Nb vs. Na	Nb vs. Nr	Nb vs. Pt	Na vs. Nr	Na vs. Pt	Nr vs. Pt
Nh vs. Nb	0.02	0.11	0.28	0.31	1.00	0.62	1.00	1.00	1.00	1.00
Pi vs. Fl	1.00	0.27	0.33	**<0.001**	0.23	0.29	**<0.001**	1.00	0.09	1.00
	**Nh vs. Nb**	**Nh vs. Na**	**Nh vs. Pt**	**Nh vs. Fl**	**Nb vs. Na**	**Nb vs. Pt**	**Nb vs. Fl**	**Na vs. Pt**	**Na vs. Fl**	**Pt vs. Fl**

We investigated if bats prefer a certain roost type/species in the flight arena depending on where they had been found in the wild. Upper part of the table: Post hoc tests for the final roost choice of bats that had been found roosting in *N. hemsleyana* pitchers (N = 16 bats) vs. *N. bicalcarata* pitchers (N = 22 bats) in the wild (global Fisher’s exact tests for count data: *P  *=0.04). Lower part of the table: Post hoc tests for those bats that finally chose a roost. These bats had been found roosting in pitchers (N = 18 bats) vs. furled leaves (N = 38 bats) in the wild (global Fisher’s exact tests for count data: *P  *<0.001). Bold values indicate significance after sequential Bonferroni correction (Abbreviation: Nh = *N. hemsleyana*, Nb = *N. bicalcarata*, Na = *N. ampullaria*, Nr = *N. rafflesiana*, Pt = Plastic tube, Pi = Pitchers, Fl = Furled leaf).

In another experiment, we tested if individuals that were found in furled leaves (*Alpinia ligulata*: 5 bats; *Boesenbergia grandis*: 3 bats; *Musa muluensis*: 6 bats) prefer one of these species. We simultaneously presented one leaf from each plant species to the bat. Except for one bat that did not choose in the end, all tested bats approached (Supplementary Results) and entered furled leaves of the different species regardless in which species we had found them roosting originally (Fisher’s exact tests for count data: *P  *=0.76; Fig. 2b).

Finally, we tested whether the bats generally prefer pitchers to furled leaves. This time, *K. hardwickii* individuals (47 captured in furled leaves, 21 in pitcher plants) could choose between one furled leaf, one *N. hemsleyana*, one *N. bicalcarata*, one *N. ampullaria* pitcher and a plastic tube. We provided several pitcher plant species but only one furled leaf as the earlier experiments (see above) had shown that the bats were not choosy when selecting furled leaves of different plant species but tended to discriminate between pitchers of different *Nepenthes* species.

Bats that originated from pitchers (individuals from all *Nepenthes* species pooled) chose *N. hemsleyana* and *N. bicalcarata* pitchers significantly more often than bats from furled leaves (all species pooled; Fig. 2c; Table 1; Supplementary Table S1). However, eight (21%) of the bats roosting in furled leaves chose pitchers during the experiment (five of these bats chose *N. hemsleyana* pitchers, one *N. bicalcarata* and two *N. ampullaria* pitchers). In contrast, not a single bat switched from pitchers to furled leaves (Supplementary Results).

### Is roost selection related to genetic differentiation?

**Figure 3:**
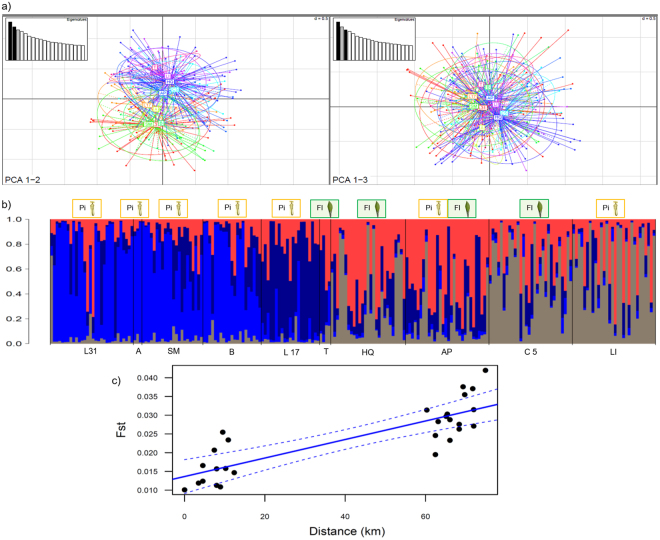
Population differentiation based on 16 microsatellite loci. (**a**) Principal component analysis (PCA) showing the axes 1–2 (axis 1 explaining 3.90% of the variance, axis 2 explaining 3.40%) and 1–3 (axis 3 explaining 3.09%) for the population structure of individuals. For each graph, the inset represents the eigenvalues of the axes. Individuals from each of 10 study sites are represented in different colours. (**b**) Results of the STRUCTURE analysis are shown for the number of populations with the best K as identified by the MedMeaK, MaxMeaK, MedMedK and MaxMedK estimators. (**c**) Relationship between genetic distance (F_ST_) and geographic distance for the eight study sites with more than five individuals/site. Habitats in which *Kerivoula hardwickii* typically occurs connect the majority of these study sites (Mantel test: r = 0.84*, P* < 0.001). The solid line represents a linear regression, dashed lines the 95% confidence interval (Abbreviation: Pi = individuals that roosted in pitchers, Fl = individuals that roosted in furled leaves; study sites Brunei Darussalam: L31 = Labi 31, A = Andulau, SM = Saw Mill, B = Badas, L17 = Labi 17, T = Teraja; study sites Sarawak/Malaysia: HQ = Headquarter, AP = Airport, C 5 = Camp 5, LI = Long Iman; images created by C. and M. Schöner).

Recent studies suggest, that *K. hardwickii* is comprised of up to five different cryptic species^23–25^, which could explain the individuals’ different roosting habits. Yet, our population genetic analyses showed low genetic differentiation between the sampled *K. hardwickii* individuals from the different study sites (pairwise F_ST_-values: mean = 0.03 ± 0.02; range: 0.001 to 0.09; Fig. 3; Supplementary Table S2), which was clearly connected to distance and not to the roost type (Fig. 3). This indicates that all individuals do belong to the same species and that it is not cryptic species, which roost in different roost types (pitchers versus furled leaves).

**Figure 4:**
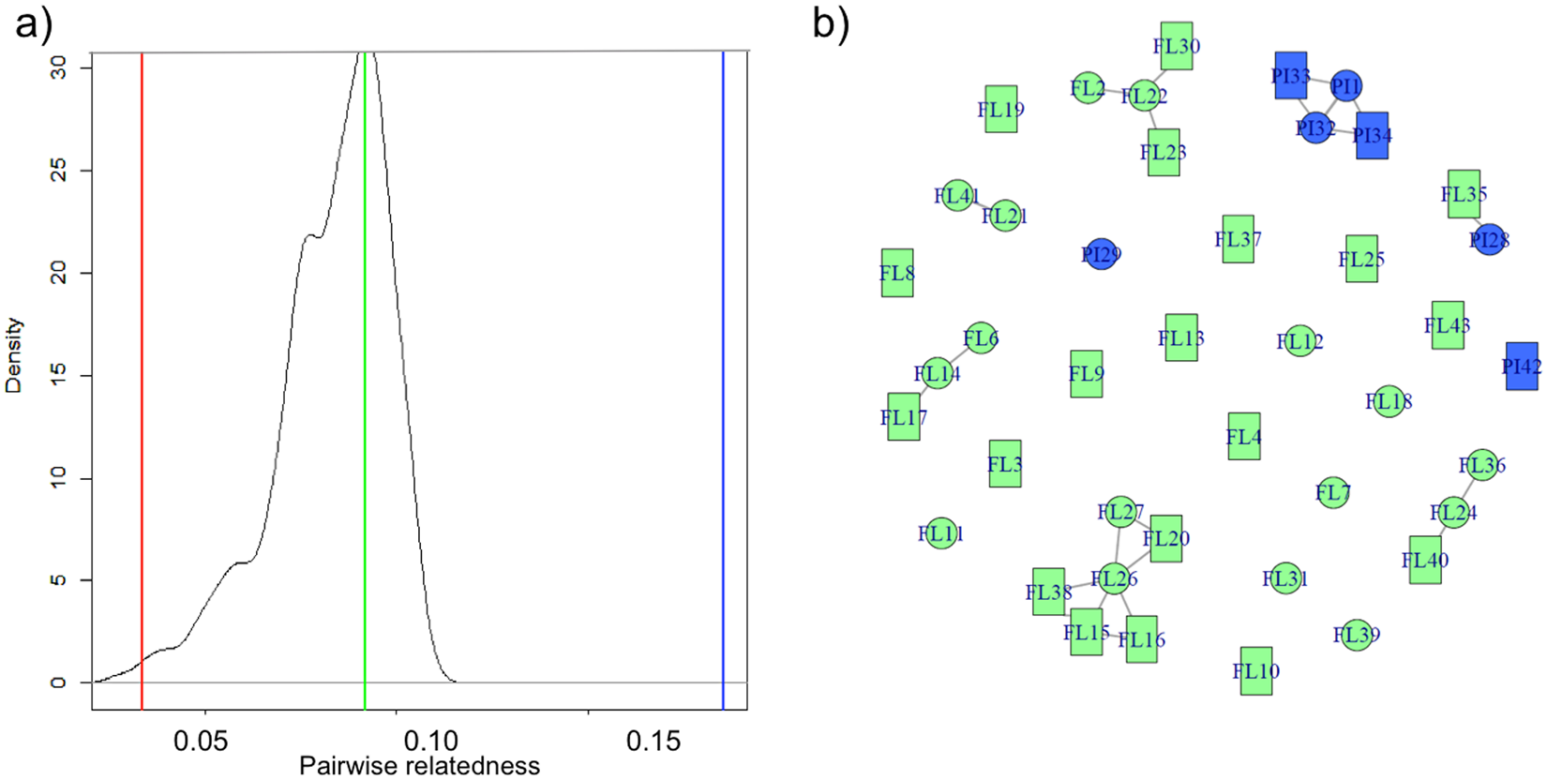
Pairwise relatedness of *Kerivoula hardwickii* at the study site “Airport”. (**a**) The graph represents mean relatedness values (TrioML, 10,000 permutations) of the seven individuals roosting in pitchers and seven randomly selected individuals roosting in furled leaves (null hypothesis distribution). Coloured lines show the mean observed relatedness for bat pairs roosting in pitchers (blue), furled leaves (green), and one bat in pitchers and the other in furled leaves (red). (**b**) Network of bats based on pairwise relatedness (TrioML). Only potential parent offspring pairs and full sibling pairs are linked (relatedness >0.44). Circles represent the females, rectangles the males, blue colour indicates bats roosting in pitchers, green those in furled leaves.

However, within the study site “Airport” pairs of bats that were both roosting in pitchers were significantly more closely related than expected by chance (Triadic Likelihood Relatedness Estimate, TrioML = 0.17 ± 0.21; Monte Carlo test: *P  *=0.004; Fig. 4) while pairs with one bat roosting in pitchers and one roosting in furled leaves were significantly less closely related than expected by chance (TrioML = 0.03 ± 0.05; *P* = 0.01). When both bats preferred furled leaves, their relatedness did not differ from random distributions (TrioML = 0.05 ± 0.10; *P  *=0.37).

## Discussion

Our results show that whenever *Nepenthes* pitchers and furled leaves were present at a given site the bats exclusively or, in one site, additionally used pitchers. During our behavioural experiments all bats that we originally had found in pitchers chose pitchers again while 21% of bats from furled leaves switched to pitchers. Quality (e.g., perceptibility or absence of parasites) is one of the most important aspects for bats to choose or to reject roosts^12,26^. As predicted by BMT^7–9^, we could already show that on an intra-specific level, *K. hardwickii* individuals select pitchers depending on certain traits, such as shape or echo-reflection^12,16–18^. The bats’ choosiness likely resulted in selection pressure on *N. hemsleyana* plants to optimize such pitcher characteristics. This should also be the case on an inter-specific level where competition for bats should have increased the selective pressure on *N. hemsleyana* plants to offer more attractive roosts than other plants. Consequently, the generally higher roosting quality of *N*. *hemsleyana* pitchers should reinforce the bats’ preference for them. Thus, the combination of *N. hemsleyana*’s high roosting quality and the bats’ choosiness should provide a unidirectional mechanism that steadily increases the number of bats using *N. hemsleyana* pitchers in areas where alternative roosting plants are available.

However, contrary to our prediction that bats will always prefer the roost with the highest quality, i.e. their mutualism partner *N. hemsleyana*, the majority of bats (79%) that originally had been roosting in furled leaves stayed faithful with this roost type. Thus, neither roost quality nor the mere abundance of the different roost types can completely explain the bats’ different roosting behaviour.

Because of *N*. *hemsleyana*’s patchiness and restricted distribution range^27^, bats cannot solely rely on that partner. Our results show, the gene flow between bat populations is maintained and genetic differences were not related to the bats’ roost use. However, on an intra-population level, individuals from study site “Airport” that shared the same roost preferences were more closely related than individuals with another roost preference. This indicates that roost preferences are likely to be transmitted between closely related individuals probably via imprinting or social learning including the formation of traditions. Konrad Lorenz defined imprinting as an individual’s behavioural response to a certain stimulus (e.g., in our study system possibly the species-specific shape or smell of a roost) to which it had been exposed during a sensitive period in early life^28^. Several animal species are imprinted to their habitats or nesting sites^29–32^. However, if imprinting would account for *K. hardwickii*’s roosting habits, the bats’ roost selection should be highly specific and stable during an individual’s life^33^, which is not the case. Neither bats roosting in furled leaves nor those roosting in pitchers were completely fixed to a certain plant species. Especially in the case of pitchers, the traits (e.g., shape, smell, etc.) of the different *Nepenthes* species used by the bats are so diverse^34^ that a general imprinting to the roost type “pitcher” seems to be unlikely.

In contrast to imprinting, social learning allows for more flexible behaviour^35^. General advantages of learning from experienced individuals can be seen in abridged learning processes compared to individual learning as juveniles can easily reproduce the behaviour of their conspecifics or learn to focus on particular cues^36^. As *K. hardwickii* is a solitary roosting bat^11^, horizontal social learning from conspecifics of the same generation can probably be neglected. Vertical social learning between generations, in contrast, is facilitated because the juveniles stay for relatively long time with their mother (at least 77 days; own observation). The bats’ faithfulness to a certain roost type could thus result from maternal social transmission that leads to different regional roosting traditions. Populations that socially transmit the preference for pitchers are reliable mutualism partners for *N. hemsleyana*. Currently, there is an on-going discussion about the influence of individual learning in pollinating insects and how this affects faithfulness to certain plant species, a behaviour called flower constancy^37,38^. In contrast, social transmission of mutualistic interactions has mostly been considered as unique for humans and their domesticated plants and animals^39^. Apart from that only few studies indicate that socially transmitted behaviours, e.g., in bumblebees, could affect mutualisms^40^.

In general, our study system shows that asymmetric power in partner choice itself can act as a stabilizing mechanism for mutualisms. First, it has already been shown that negative biotic and abiotic influences can lead to a cascade that ends in co-extinction of both interacting species when the partners completely rely on each other. Thus, in mutualistic networks, highly specialised partners often rely on more generalist partners with less partner-specific individuals that may prevent coextinction of both species^41–44^. Second, the asymmetry increases the selective pressure on the partner with lower power in partner choice to offer high-quality commodities. *Nepenthes hemsleyana* plants do not only face intraspecific competition for *K. hardwickii* bats (as it would be the case in an obligatory mutualism) but also interspecific competition that may affect various traits of the pitchers such as their shape, microclimate or longevity, and may lead to pronounced adaptations.

In conclusion, the mutualism between bats of the species *K. hardwickii* and *N. hemsleyana* plants seems to be stabilized by a combination of two mechanisms: 1) the bats’ general preference for high quality roosts and 2) asymmetric power in partner choice with socially transmitted roost preferences of the bats. Thus, we encourage ecologists to further investigate the influence of social transmission on diverse mutualisms – a phenomenon that is probably widespread in interspecific interactions.

## Methods

### Study periods and study sites

Bats were caught in harp traps or in their roosts (*Nepenthes* pitchers and furled leaves of the plant families Zingiberaceae, Musaceae, Araceae) in the Belait district of Brunei Darussalam and in the Mulu National Park in Sarawak/Malaysia during four field seasons (from 14 June to 30 July 2009, from 14 August 2011 to 14 January 2012, from 20 June to 3 December 2012 and from 14 April to 1 September 2014; see Supplementary Table S1). All adult males and non-reproductive females were marked with PIT-tags (ISO 11784/11785; Peddy-Mark, UK) for individual identification^12^.

### *Kerivoula hardwickii*’s roost choice

#### Field observations

In each study site we monitored the occurring *K. hardwickii* individuals for 30.0 ± 18.3 days (mean ± s.d.) by daily checking all potential roosts (furled leaves and *Nepenthes* pitchers below a height of 2.5 m) and additionally by catching individuals with harp traps. We radio-tracked on average 5.5 ± 3.8 (range: 0–12) individuals per site. Parts of the radio-tracking data have already been published^11,12^. Additionally, individuals could easily be identified from outside the roost with a handheld PIT-tag reader (LID-575 Midrange Reader, Trovan, UK). Of special interest to us was study site “Airport” where bats not only use pitchers of the species *N. hemsleyana and N. bicalcarata* but also furled leaves (*M. muluensis, Zingiber kelabitianum*, *Plagiostachys albiflora*, *Plagiostachys strobilifera*) as roost. In this study site we radio-tracked three *K. hardwickii* individuals (two males, one female) from furled leaves and one male individual from a pitcher for an overall mean of 8.50 ± 2.87 days.

#### Experimental set-up

In a flight arena (length and width 3.5 m, height 2.5 m, Fig. 1) we conducted three types of behavioural experiments where we investigated if bats generally prefer a certain plant species, if they prefer the species in which we had found them or if they randomly choose between different plant species.

In all experiments, we randomly arranged pitchers and furled leaves within the flight arena (distance to each other = 0.5 m; height = 1.5 m). To prevent the plants from excessive damage by cutting pitchers and leaves, we offered the same experimental leaf/pitcher to up to three bats. We tested each bat only once per experiment and released them within 24 hours of capture into their original habitat. Before and after the experiment we fed the bats with mealworms and offered them water ad libitum. We excluded pregnant and lactating females as well as juveniles.

We filmed (Sony HDR-CX560VE) all experiments to determine how the bats react to different roost types (pitchers or furled leaves) or plant species, how often they approach to the different potential roosts and which of them they finally choose. We defined an approach as hovering flight in front of an object within a distance of 10 cm. Three bats in the first, one bat in the second and twelve bats in the last experiment did not choose a roost within the maximum time span of 30 min per trial and thus were excluded from the analyses of the bats’ final roost selection.

First, we simultaneously offered one *N. hemsleyana*, one *N. bicalcarata*, and one *N. ampullaria* pitcher, and additionally a pitcher of *N. rafflesiana*, which is not used by the bats, as well as a plastic tube (Fig. 2a). We tested 41 bats (12 males, 29 females) from areas where *K. hardwickii* only used pitchers as roosts, although furled leaves were available. Sixteen individuals derived from an area where the bats exclusively use *N. hemsleyana* pitchers, the other bats were captured at study sites where the bats exclusively roost in *N. bicalcarata* pitchers or where they use both pitcher plant species (see Table S1 for roost availabilities per plant species and site). In the latter case, we only tested individuals that exclusively roosted in *N. bicalcarata* pitchers during a radio-tracking period of 5–13 days (9.82 ± 2.64 days; for details see^12^).

Similarly, in a second experiment we tested 14 bats (10 males, 4 females) that had roosted in furled leaves (Fig. 2b). For the experiment we simultaneously offered a total of three furled leaves, one of each species: *A. ligulata*, *B. grandis*, and *M. muluensis*.

Finally, we tested how 68 bats (56 males, 12 females) reacted to different roost types (pitchers versus furled leaves). We offered one *N. hemsleyana*, one *N. bicalcarata*, and one *N. ampullaria* pitcher, one furled leaf (*A. ligulata*, *B. grandis* or *M. muluensis*; for the 47 bats that we had found in furled leaves we used the respective roost plant species; in the case of the 21 bats found in pitchers we used the plant species that occurred within a distance of 20 m from the roost) as well as the plastic tube as roost (Fig. 2c).

### Statistical data analysis

We compared the distribution of observed approaches to permutated datasets in which observed approach numbers were randomly allocated to the three/five provided roost types following the approach used in^17^. For the Monte Carlo tests, we tested the null hypothesis that the roost type did not affect the number of approaches. We first calculated the mean number of approaches for each roost type, which we then compared to the distribution of values expected under the null hypothesis. We obtained the null hypothesis distribution by permuting the number of approaches between roost types for each tested animal and then calculating the mean number of approaches per roost type. To obtain the null distribution of the mean number of approaches we repeated this procedure 10,000 times. Then we calculated the *P*-value by comparing the mean number of approaches for the considered roost type to the null distribution.

### Genetic analysis

#### Sample collection and DNA extractions

We took samples with a sterile biopsy punch (Stiefel Laboratories; diameter: 2 mm) of 317 bats from 10 locations (six in Brunei Darussalam, four in Sarawak). Samples were stored in 90% ethanol or dried with silica gel until DNA extraction (Silica Gel Orange, Carl Roth GmbH)^45^. DNA was extracted from wing biopsy punches using a modified ammonium acetate extraction protocol (Strauss 2001), eluted in Low TE and stored at −20 °C. We used DNA samples at final concentrations of at least 2 ng μl^−1^ (quantified from extracted samples on a NanoDrop ND-1000 Spectrophotometer, Thermo Fisher Scientific).

#### Microsatellite development

We sent genomic DNA to the Max-Planck-Institute for Evolutionary Biology in Plön that created a microsatellite library using high-throughput shotgun 454-sequencing. Using the programme MISA (Microsatellite Identification Tool; http://pgrc.ipk-gatersleben.de/misa/misa.html) we found 66,289 potential microsatellite sequences from which we developed 40 unlabelled primer pairs using the programmes Nucleic Acid Sequence Massager (http://www.attotron.com/cybertory/analysis/seqMassager.htm) for cleaning the sequences and Primer 3 v. 4.0.0 (http://sourceforge.net/projects/primer3/)^46,47^ to design the primers. Using pooled DNA from two individuals we tested these primer pairs for amplification and polymorphism at four different annealing temperatures (56–62 °C; ABI 3130xl Genetic Analyser, Applied Biosystems).

#### Microsatellite amplification and data analysis

Based on amplification success and polymorphism, a set of 16 loci was selected (Supplementary Table S3) and optimised in two multiplex reactions (MP1/MP2) which were conducted for each individual in 8 μl (MP1) and 5 μl (MP2) reaction volumes, each consisting of 1.0 μl DNA, 1 × Multiplex PCR Master Mix (Qiagen) and primer concentrations as indicated in Table S3. We used the following amplification conditions: 95 °C for 15 min; 32 cycles of 94 °C for 30 s, 60 °C for 90 s, 72 °C for 60 s; 60 °C for 30 min. All PCR products were run on an ABI 3130xl Genetic Analyser (Applied Biosystems) and sized with an internal lane standard (GeneScan™ 500 LIZ™ dye Size Standard, Thermo Fisher) and the software GeneMapper v. 5 (Applied Biosystems).

To check for genotyping consistency, 23.0% of samples were amplified and genotyped twice. We could not detect departures from Hardy-Weinberg and linkage equilibrium at the site level after Bonferroni correction using Genepop v. 4.1.4 (except for individuals of study site “Labi 31” where we had 33 significant linkages between markers probably due to the presence of close relatives). We also found no evidence for the presence of null alleles, large allelic drop-out or possible scoring errors across populations within our dataset (tested with Micro-checker v. 2.2.3)^48^.

To investigate if there is a correlation between the populations’ pairwise genetic distance and pairwise geographic distance matrices, we conducted a Mantel test (99,999 permutations) with the R package ecodist^49^. We calculated F_ST_ with GenoDive v.2. Ob27 to measure pairwise population differentiation. With STRUCTURE v. 2.3.4^50,51^ we investigated the population structure using a burn-in length of 20,000 and a run length of 200,000 without prior population information. The admixture model and the correlated allele frequencies between population options were selected. After an initial test we chose the burn-in and run length by looking at the convergence of the values of summary statistics and consistency between runs. All other parameters were left as by default. We undertook ten independent runs for K-values ranging from one to ten, which reflects the minimum and maximum number of populations suspected. The number of populations was inferred from the corrected posterior probability and four new estimators that have been shown to outperform other estimators, namely MedMeaK, MaxMeaK, MedMedK and MaxMedK^52^. Additionally, we conducted a Principal Component Analysis (based on individuals) using the adegenet v. 1.3-9^53^ and ade4 v. 1.4–14^54^ packages in R (Fig. 3).

For all bats of the study site “Airport” (which is the only study site where bats were roosting in pitcher plants and in furled leaves) we calculated pairwise relatedness (triadic likelihood relatedness estimate (TrioML) with Coancestry v. 1.0.1.5^55^. With Monte Carlo we tested the null hypothesis that the pairwise relatedness of bats did not differ in relation to the preferred roost type (pitchers, furled leaves). Therefore, we randomly selected (1,000 times) seven individuals roosting in furled leaves and combined them with the seven individuals roosting in pitcher plants. We compared the mean pairwise relatedness of bat pairs roosting in pitchers, of bat pairs roosting in furled leaves, and of bat pairs with differing roost preference (one in pitchers, one in furled leaves) to the distribution of values expected under the null hypothesis. We obtained the null hypothesis distribution by randomly assigning roost preferences and then calculating mean difference for pairs roosting in furled leaves, in pitchers, or in both. This procedure was repeated 10,000 times. To calculate the *P*-values, we compared the observed mean values of relatedness to the null distributions. To visualize the observed pairwise relatedness (TrioML) between the individuals at the study site “Airport”, we constructed an unweighted and undirected network of the bats using the R package igraph v. 0.7.1^56^. To focus on very closely related pairs of bats (parent-offspring or full-sibling pairs), we kept only links with TrioML relatedness >0.44 (Fig. 4).

### Ethical Statement

All procedures performed with bats were carried out in accordance to the Animal Behaviour Society^57^ and were approved by the University Brunei Darussalam Research Committee (UBD/PNC2/2/RG105 &193) and the Forestry Department Sarawak (NCCD.907.4.4(JLD.10)-209, (JLD.12)-20 and NO. 173/2014) that gave permission to capture and handle the bats.

### Data availability

Microsatellite sequences for *Kerivoula hardwickii* are available via the following GenBank accession numbers: MF919263, MF919265, MF919305, MF919271, MF919277, MF919279, MF919309, MF919293, MF919294, MF919296, MF919301, MF919268, MF919307, MF919285, MF919287, and MF919310 (BankIt2046125). Data are made available in the Supplement or can be requested from the corresponding author.

## Electronic supplementary material

Supplementary PDF File

Supplementary DOC File
