# Supplementary material for: Stabilization of a bat-pitcher plant mutualism
Source: Sci Rep. 2017 Oct 13;7:13170. doi: 10.1038/s41598-017-13535-5 (PMC5640698; doi:10.1038/s41598-017-13535-5)
Supplement: Supplementary file 1 — Supplementary PDF File [file 41598_2017_13535_MOESM1_ESM.pdf]

Michael G. Schöner, Caroline R. Schöner, Rebecca Ermisch, Sébastien J. Puechmaille, T. Ulmar Grafe, Moi Chan Tan, Gerald Kerth

Stabilization of a bat-pitcher plant mutualism

Supplement

Supplementary Results

Behavioural experiments

When the bats could select between pitchers of different *Nepenthes* species, bats originally roosting in *N. hemsleyana* pitchers approached *N. hemsleyana* pitchers more often than expected by chance while they approached *N. rafflesiana* significantly less often than expected by chance (Figure S1d). In contrast, bats from *N. bicalcarata* pitchers randomly approached all potential roost species (Figure S1e). When bats could choose between pitchers of different species and a furled leaf, bats from furled leaves approached more often to furled leaves and visited *N. bicalcarata* pitchers less frequently (Figure S1f) while bats from pitchers significantly more often approached *N. hemsleyana* pitchers but less often furled leaves and the plastic tube (Figure S1e).

To correct for the higher proportion of pitchers compared to the single furled leaf (3 : 1), we divided the number of bats that selected pitchers in the flight arena by three (assuming equal preference for all pitchers). Still, bats clearly preferred their original roost type to the unfamiliar one: Only 2.67 of the 8.67 bats that chose pitchers derived from furled leaves while none of the 29 bats that chose furled leaves derived from pitchers (Fisher's exact test for count data: $P < 0.0001$).

Genetic analysis of the different populations

Between study sites, pairwise F_{ST}-values (mean = 0.03 ± 0.02; range: 0.01 to 0.09; Table S2) showed low population differentiation and differentiation seemed to be independent of the bats' roost preference. Rather than bat roost preference, population structure was linked to geography. We identified three clusters within our ten sampling locations/populations: Cluster 1) "Labi 31", "Andulau", "Saw Mill", "Badas" (Brunei); Cluster 2) "Labi 17", "Teraja" (Brunei); Cluster 3) "Camp 5", "Airport", "Headquarter", "Long Iman" (Sarawak/Malaysia; Figure 3b). Clusters 2) and 3) comprise both bats roosting in pitchers and bats roosting in furled leaves. Similarly, a PCA analysis showed no clear differentiation between bats roosting in pitchers and those in furled leaves (Figure 3a). However, there was a significant relationship between geographic and genetic distance (F_{ST}) regarding the 10 sampling sites (r = 0.29, P = 0.009; Figure 3c).

Roost choice and its effect on the relatedness of the bats

Although in six of the 10 study sites the monitored bats used different roost species, they never used both, pitchers and furled leaves, except in the study site "Airport". Here we monitored 42 bats for a mean time period of 3.76 ± 3.24 days. On an individual level, however, the bats did not switch but either used pitchers (seven bats roosted in 12 *N. hemsleyana* pitchers, two of them additionally in three *N. bicalcarata* pitchers) or furled leaves (35 bats roosted in 136 *M. muluensis* plants, which provided 79.40 % of all furled leaves, one of these bats switched between furled leaves of *M. muluensis*, *Z. kelabitianum*, *P. albiflora*, *P. strobilifera* but used each of the latter three species just for one day). We found 21 potential parent-offspring pairs or full-siblings (TrioML > 0.45). All pairs roosted in the same roost type except of one, which involved a male and a female (Chi-squared test for given probabilities: χ^2 = 17.19, df = 1, P < 0.0001; Figure 4).

Supplementary Figure S1: Approaches of *Kerivoula hardwickii* to potential roosts. a)-c) 14 bats found roosting in furled leaves in the wild could choose between furled leaves of different plant species (*Alpinia ligulata* (Al), *Boesenbergia grandis* (Bg), *Musa muluensis* (Mm)). d)-e) 41 bats found roosting in pitchers of *Nepenthes hemsleyana* or *Nepenthes bicalcarata* could choose between pitchers of different *Nepenthes* species (*N. hemsleyana* (Nh), *N. bicalcarata* (Nb), *Nepenthes ampullaria* (Na), *Nepenthes rafflesiana* (Nr)) and a plastic tube (Pt). f)-g) 21 bats found roosting in pitchers and 47 bats found in furled leaves in the wild could choose between a furled leaf, three pitchers (Nh, Nb, Na) and the plastic tube. For statistics see Supplementary methods. Red colour indicates significant *P*-values after sequential Bonferroni correction.

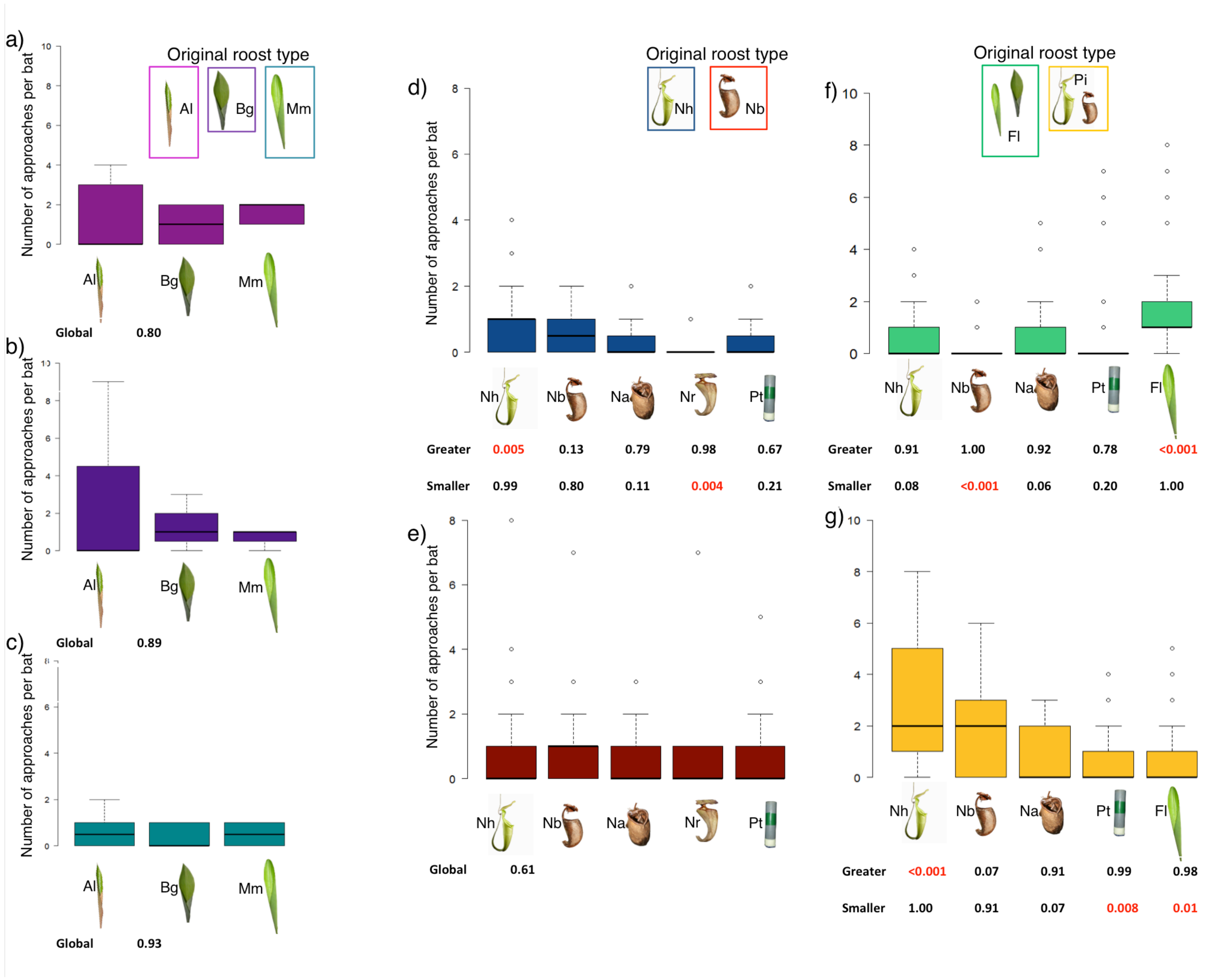

Supplementary Table S1: Study sites, available and occupied roost types and monitored roosts and bats. Percentages indicate the share of roosts of a given type/species that were available. Percentages in brackets indicate the share of chosen roosts in a study site. Furled leaves were available in all study sites and checked for bats although we did not quantify them in all sites (indicated by "n.a."). In the table we did not distinguish between furled leaves of different plant species as the bats showed no significant preferences for one of the furled leaves' species.

Study site	Occuring (and occupied) *Nepenthes* species	Occuring (and occupied) furled leaves	Total monitoring time [days]
**Labi 31, Brunei**	*N. hemsleyana*: 86% (100%)<br>*N. bicalcarata*: 12% (0%)<br>*N. ampullaria*: 2% (0%)	n.a. (0%)	65
**Labi 17, Brunei**	*N. hemsleyana*: 13% (26%)<br>*N. bicalcarata*: 87% (74%)	n.a. (0%)	41
**Andulau, Brunei**	*N. hemsleyana*: 100 % (100%)	n.a. (0%)	19
**Saw Mill, Brunei**	*N. hemsleyana*: 11% (24%)<br>*N. bicalcarata*: 46% (76%)<br>*N. ampullaria*: 38% (0%)	n.a. (0%)	36
**Badas, Brunei**	*N. hemsleyana*: 3% (0%)<br>*N. bicalcarata*: 69% (100%)<br>*N. ampullaria*: 28% (0%)	n.a. (0%)	43
**Teraja, Brunei**	0% (0%)	100% (100%)	12
**Headquarter, Sarawak/Malaysia**	*N. hemsleyana*: 1% (0%)<br>*N. ampullaria*: 1% (0%)	98% (100%)	37
**Airport, Sarawak/Malaysia**	*N. hemsleyana*: 4% (9%)<br>*N. bicalcarata*: 6% (3%)<br>*N. ampullaria*: 11% (0%)	79% (88%)	37
**Camp 5, Sarawak/Malaysia**	0% (0%)	100% (100%)	5
**Long Iman, Sarawak/Malaysia**	*N. hemsleyana*: 5% (21%)<br>*N. ampullaria*: 57% (79%)	38% (0%)	5

Supplementary Table S2: F_{ST} values of the different monitored *Kerivoula hardwickii* populations (lower part of the matrix:) and corresponding *P*-values for all pairs of populations (upper part of the matrix). Bold values indicate significance after sequential Bonferroni correction.

	Labi 31	Andu -lau	Saw Mill	Badas	Labi 17	Teraja	Camp 5	Air-port	Head-quarter	Long Iman
**Labi 31**	-	0.024	**0.001**	**0.001**	**0.001**	0.009	**0.001**	**0.001**	**0.001**	**0.001**
**Andulau**	0.037	-	0.017	0.008	0.072	0.038	**0.001**	**0.001**	**0.001**	**0.001**
**Saw Mill**	0.013	0.052	-	0.020	0.021	0.011	**0.001**	**0.001**	**0.001**	**0.001**
**Badas**	0.017	0.058	0.011	-	0.026	0.008	**0.001**	**0.001**	**0.001**	**0.001**
**Labi 17**	0.025	0.007	0.003	0.001	-	0.090	**0.001**	**0.001**	**0.001**	**0.001**
**Teraja**	0.035	0.091	0.044	0.043	0.020	-	0.024	0.020	0.020	0.023
**Camp 5**	0.028	0.06	0.029	0.031	0.025	0.038	-	**0.002**	**0.001**	**0.001**
**Airport**	0.027	0.064	0.023	0.027	0.019	0.061	0.010	-	**0.001**	**0.001**
**Head-quarter**	0.030	0.063	0.028	0.035	0.031	0.039	0.011	0.016	-	**0.001**
**Long Iman**	0.037	0.065	0.038	0.042	0.030	0.033	0.012	0.017	0.015	-

Supplementary Table S3: Sequences and characteristics of the used microsatellite primers. Abbreviations: F = forward primer, R = reverse primer, H_O = observed heterozygosity, H_S = heterozygosity within populations, H_T = total heterozygosity

Locus	Inheritance	Repeat motif	Primer sequence (5‘-3‘)	Primer (µM)	Size range (bp)	n	H_0	H_S	H_T
H4LQB	autosomal	$(AC)_{14}$	F: NED-TACTGAAGGCCCTGGGAAG	0.625	223-255	15	0.867	0.859	0.879
			R: GTTT-GGGAACACCTGATACATGCTAAG						
H5ASK	autosomal	$(TG)_{18}$	F: FAM-CCAGCTTGTCCCATCTTACAC	0.625	172-206	17	0.903	0.882	0.897
			R: GTTT-GCTATGAGCCTCCAAACTGC						
H709A	autosomal	$(AG)_{11}$	F: FAM-GCCCATGAACTTTGCATCTTAC	0.250	110-130	11	0.480	0.498	0.520
			R: GTCACAATCCCTGCCAGTTC						
H7V93	autosomal	$(AG)_{10}$	F: FAM-TCTTCCTTAATGGCAGGACTTC	0.625	235-266	14	0.821	0.842	0.875
			R: GTTT-GGAGGTCAGGGTTCAATTCTC						
HU2ND	autosomal	$(CA)_{16}$	F: VIC-GCTTGGCAAACCATCACC	0.375	105-142	22	0.871	0.849	0.879
			R: GGCTCTGAATGTGGGTTCAC						
HV644	autosomal	$(CA)_{10}$	F: VIC-CGCCAGCAGATCCTAGAGAC	0.625	236-258	11	0.777	0.728	0.775
			R: GTTT-CCTCGATCTAACCACTGTATTTGAC						
IGALM	autosomal	$(AC)_{11}$	F: PET-CCATAAGAGGGAGGAATGAGG	0.250	98-118	9	0.655	0.669	0.658
			R: GTTCAGCATGAGTGTATATGAGTGTG						
IKOZA	autosomal	$(AC)_{11}$	F: PET-TGCCACAATCACACATTCTATG	0.375	279-289	6	0.695	0.696	0.735
			R: AGGTCTGGAGCAAAGACACTTC						
ILV82	autosomal	$(AC)_{15}$	F: VIC-CTCACGCTACTCCAGGAAGG	0.375	172-198	11	0.844	0.817	0.843
			R: GTT-TAACATCTGCCATGTACCCATC						
INWUN	autosomal	$(TG)_{13}$	F: PET-ACTGGGCAATGTCCAAAGAC	0.625	178-210	28	0.935	0.926	0.952
			R: GT-TTCTTTGCTGTGGGAGCAG						
H0VJJ	autosomal	$(GT)_{14}$	F: VIC-TCCTCATCAAGATATGAACATTGG	0.250	133-151	9	0.851	0.793	0.807
			R: GTT-TCAAGAAAGTGAGCTATGAAGCAG						
H6XP6	autosomal	$(AC)_{17}$	F: FAM-AATGAGACAGCAAAGCAAGAAAC	0.375	188-220	17	0.943	0.890	0.907
			R: AGTTGGATTCCCAGTCATGG						
HZIGX	autosomal	$(AC)_{15}$	F: FAM-TCTATCTCCAATGTAACTCCAAAGC	0.375	268-290	16	0.801	0.794	0.854
			R: GTGCCAGAAGCATCTGCTAAG						
IDH61	autosomal	$(AC)_{14}$	F: VIC-GGCTCAAATTGTGCTAAATGG	0.250	203-237	17	0.814	0.806	0.825
			R: GTT-TACTGGGTGGCTGCAGAAG						
IEP4F	autosomal	$(AC)^{17}$	F: PET-TTCCGGAAGAGTCTAGGATGG	0.625	232-262	19	0.893	0.903	0.918
			R: CGCACTGTCCAATCTCAGG						
IK4V2	autosomal	$(AC)_{20}$	F: FAM-CAAGCCTCTTATGCAACTAGGG	0.250	94-121	20	0.866	0.864	0.886
			R: GTT-TGCCTGTATCTGGGAGCAG						
